# Urinary miRNA profile for the diagnosis of IgA nephropathy

**DOI:** 10.1186/s12882-019-1267-4

**Published:** 2019-03-04

**Authors:** Cheuk-Chun Szeto, Gang Wang, Jack Kit-Chung Ng, Bonnie Ching-Ha Kwan, Fernand Mac-Moune Lai, Kai-Ming Chow, Cathy Choi-Wan Luk, Ka-Bik Lai, Philip Kam-Tao Li

**Affiliations:** 1 Department of Medicine & Therapeutics, Prince of Wales Hospital, The Chinese University of Hong Kong, Shatin, NT, Hong Kong China; 2 Department of Anatomical & Cellular Pathology, Prince of Wales Hospital, The Chinese University of Hong Kong, Shatin, Hong Kong; 3 Division of Nephrology, The First Affiliated Hospital of Shenzhen University, Shenzhen, China

**Keywords:** Glomerulonephritis, Biomarker, Immunology, Inflammation, Chronic kidney disease, Proteinuria

## Abstract

**Background:**

IgA nephropathy (IgAN) is the most common primary glomerulonephritis worldwide. Urinary micro-RNA (miRNA) level is increasingly reported to as non-invasive markers of various kidney diseases. We aim to identify urinary miRNA targets for the diagnosis of IgAN.

**Methods:**

In the development cohort, we performed complete miRNA profiling of urinary sediment in 22 patients with IgAN and 11 healthy controls (CTL). Potential miRNA targets were quantified by a separate validation cohort of 33 IgAN patients and 9 healthy controls.

**Results:**

In the development cohort, we identified 39 miRNA targets that have significantly different expression between IgAN and CTL (14 up-regulated, and 25 down-regulated). Among the 8 miRNA targets chosen for validation study, urinary miR-204, miR-431 and miR-555 remained significantly reduced, and urinary miR-150 level was significantly increased in the IgAN as compared to CTL. The area-under-curve of the receiver operating characteristic (ROC) curve for urinary mi-204 level for the diagnosis of IgAN was 0.976, and the diagnostic performance of combining additional miRNA targets was not further improved. At the cut-off 1.70 unit, the sensitivity and specificity of urinary miR-204 was 100 and 55.5%, respectively, for diagnosing IgAN.

**Conclusions:**

Urinary miR-150, miR-204, miR-431 and miR-555 levels are significantly different between IgAN and healthy controls; urinary miR-204 level alone has the best diagnostic accuracy.

**Electronic supplementary material:**

The online version of this article (10.1186/s12882-019-1267-4) contains supplementary material, which is available to authorized users.

## Background

Immunoglobulin A nephropathy (IgAN) is the most common primary glomerulonephritis in the world [1]. The clinical course of IgAN, however, is highly variable. Around one-third of the patients progress to dialysis-dependent renal failure in 10 years [2], while many others remain asymptomatic despite persistent abnormal urinalysis [3]. At present, the major clinical tools for predicting prognosis of IgAN are baseline renal function, proteinuria, and histological grading [2–4]. Non-invasive biomarkers are much needed for risk stratification as well as monitoring of response to treatment.

MicroRNA (miRNA) is a group of short noncoding RNA molecules with a length around 22 bases that regulates gene expression at the post-transcriptional level by incomplete base pairing with the 3′-untranslated region of target messenger RNAs [5]. In the context of IgAN, a genome-wide analysis of peripheral blood mononuclear cell miRNA profile showed that abnormal miR-148b expression in B lymphocyte may be responsible for the aberrant glycosylation of IgA1, and plays a central role in the early phase of pathogenesis [6]. On the other hand, intra-renal miR-29c level is important for the regulation of renal fibrosis and later stages of IgAN progression [7].

Recently, miRNA level in urinary sediment has been explored as non-invasive biomarkers of kidney diseases [8–10]. A few early studies suggest that urinary levels of several miRNAs are substantially changed in IgAN [11]. For example, urinary miR-200a, miR-200b and miR-429 levels were reported to be decreased in IgAN [12], while urinary miR-29b, miR-29c, and miR-93 levels correlated with renal function and the severity of histological damage [13]. Published data on urinary miRNA, however, are scattered and focused on target miRNAs. In the present study, our objective is to identify a panel of urinary miRNA targets that could be used for the diagnosis and risk stratification of IgA nephropathy.

## Methods

### Overall study design

This is an observational study approved by the Clinical Research Ethics Committee of the Chinese University of Hong Kong. All study procedures were in compliance with the Declaration of Helsinki. For the development cohort, we recruited 22 patients with biopsy-confirmed IgAN and 6 healthy volunteers as controls (CTL). For the validation cohort, we recruited 33 patients with biopsy-confirmed IgAN and 9 healthy volunteers as controls (CTL). For the IgAN group, we excluded patients with crescentic changes in the kidney biopsy, IgA vasculitis, or concomitant urinary tract infection.

After written informed consent, a whole-stream early morning urine specimen was collected for RNA extraction. We reviewed the patients’ demographic, clinical, and pathological information. Clinical parameters reviewed included proteinuria, estimated glomerular filtration rate (GFR) as estimated by a standard equation [14].

### miRNA extraction

RNA isolation kit was purchased from Ambion, Inc. Austin, TX, USA. The methods of RNA extraction from urine have been described previously [15]. Briefly, urine specimens were collected and sent to laboratory for processing immediately or stored in 4 °C overnight. Urine samples were centrifuged at 3000-g for 30 min and at 13000-g for 5 min at 4 °C. Specimens were then be stored at − 80 °C until use.

### NanoString miRNA assay

The digital multiplexed NanoString nCounter miRNA Expression Assay (NanoString Technologies, Seattle, WA) was performed according to manufacturer’s instructions with total RNA extracted [16]. Briefly, we prepared miRNA by ligating a specific tag onto the 3′ end of each mature target, followed by hybridization at 65 °C overnight to nCounter Reporter and Capture probes. Residual Reporter and Capture probes were removed by an automated station. Bound complexes were then immobilized in standard cartridges, which were then placed in a digital analyzer for data acquisition. The nSolver Analysis software (NanoString) (version 2.5) was used for data analysis. Gene counts were control-normalized and compared to that of β2-microglobulin.

### Quantification of specific miRNA

For the validation cohort, urinary level of RNA targets identified in the previous part were measured by real-time quantitative polymerase chain reaction (RT-QPCR) using the ABI Prism 7900 Sequence Detection System (Applied Biosystems, Foster City, CA, USA). We used commercially available unlabeled Taqman® primers and FAM™ dye-labeled TaqMan® MGB probe for the targets (Applied Biosystems), and the RNU48 as the house-keeping gene [10, 11]. We analyzed the results by Sequence Detection Software version 2.0 (Applied Biosystems). For quantitation, we used the relative ΔΔCT method. As described previously [17, 18], when there was no detectable transcript after 40 cycles, we assigned the value half of the detection limit for non-parametric analysis.

### Histological assessment

Histological lesions were classified by the revised Oxford classification [19]. The severity of glomerulosclerosis and tubulointerstitial fibrosis were further assessed by morphometric study as described previously [20, 21]. Briefly, Jones’ silver staining was performed on 4 μm thick sections of renal biopsy specimen. Computerized image analysis method was then performed by the MetaMorph 4.0 image-analyzing software (Universal Imaging Corporation™, Downingtown, PA). Ten glomeruli and 10 randomly selected tubulointerstitial areas were assessed for each patient.

### Clinical follow up

We further reviewed the progression of renal function during subsequent follow up. The slope of decline of estimated GFR was calculated by the least squares regression method. Renal events were defined as 30% worsening of estimated GFR as compared to baseline or the need of dialysis.

### Statistical analysis

Statistical analysis was performed by SPSS for Windows software version 24.0 (IBM corporation, Armonk, NY). Data were expressed as mean ± standard deviation or median (inter-quartile range [IQR]) as appropriate. The levels of individual miRNA targets were compared by Mann Whitney U test between groups, and the correlation with clinical parameters were explored by the Spearman’s rank correlation coefficient. Receiver operating characteristic (ROC) curves were constructed by standard methods. *P* value below 0.05 was considered statistically significant. All probabilities were two-tailed.

## Results

### Characteristics of study participants

The clinical and demographic characteristics of patients participating in this study are summarized in Table 1. The pathological characteristics of the IgAN group are summarized in Table 2.

**Table 1:** Demographic and baseline clinical data

group	development cohort		validation cohort	
	IgAN	CTL	IgAN	CTL
no. of subjects	22	6	33	9
sex (M:F)	3:19	2:4	10:23	4:5
age (year)	46.5 ± 15.9	37.3 ± 4.7	45.1 ± 10.2	37.4 ± 4.0
proteinuria (g/day)	2.1 ± 2.1	–	2.0 ± 0.9	–
serum creatinine (μmol/l)	155.0 ± 214.7	–	136.6 ± 79.8	–
estimated GFR (ml/min/1.73m^2^)	66.7 ± 31.0	–	60.2 ± 28.4	–

*IgAN* IgA nephropathy, *CTL* healthy control, *GFR* glomerular filtration rate

**Table 2:** Pathological characteristics of patients with IgA nephropathy

	development cohort	validation cohort
no. of subjects	22	33
glomerulosclerosis (%)	18.5 ± 17.3	21.6 ± 22.2
tubulointerstitial fibrosis (%)	15.3 ± 16.3	21.3 ± 24.2
Oxford classification		
mesangial score		
M0	11	17
M1	11	16
endocapillary hypercellularity		
E0	20	29
E1	2	4
segmental glomeruloscerlosis		
S0	12	18
S1	10	15
tubular atrophy / interstitial fibrosis		
T0	17	18
T1	3	8
T2	2	7
cellular / fibrocellular crescents		
C0	21	33
C1	1	0
C2	0	0

### Urinary miRNA level in the development cohort

**Fig. 1:**
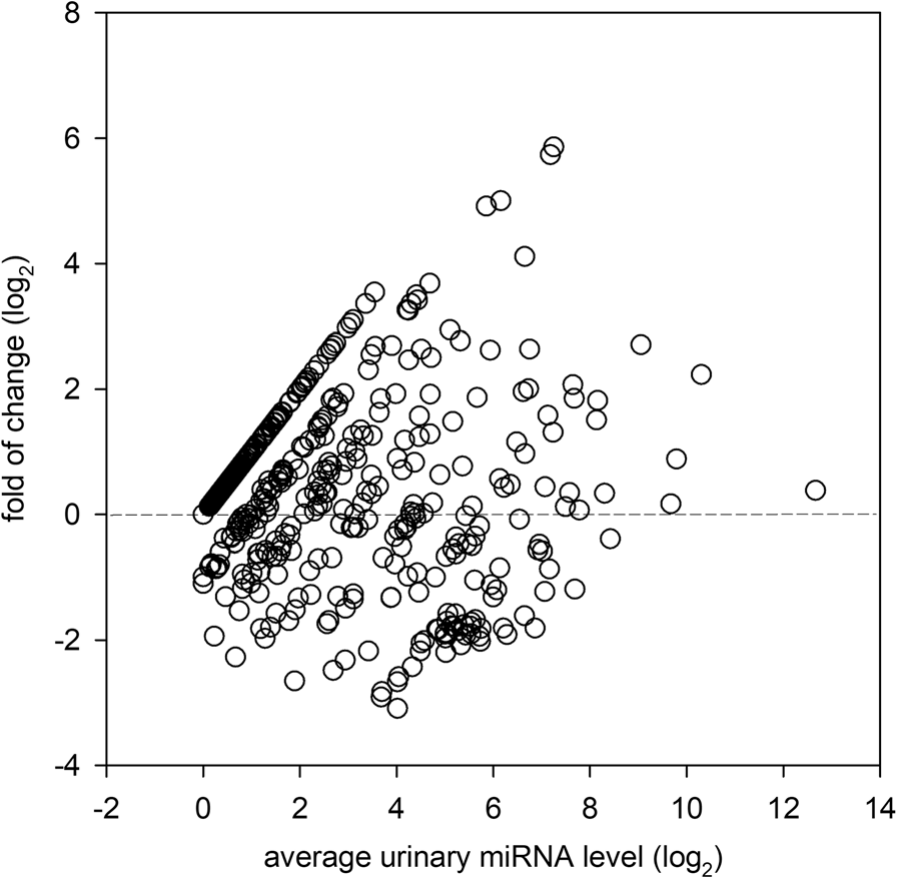
Scatter plot depicting the distribution and correlation between average urinary miRNA of the IgA nephropathy (IgAN) group and the change in urinary miRNA levels (IgAN as compared to the control group) in the development cohort. Urinary miRNA levels were compared to that of β2-microglobulin (see *Patients and Methods* for details). The change in urinary miRNA levels is presented as log_2_ of the ratio between average urinary miRNA levels of the IgAN to the CTL group. Each dot represents one miRNA target

The correlation between the average urinary miRNA level and the fold of change in urinary miRNA level between IgAN and CTL groups is depicted in Fig. 1. Among the miRNA targets that are highly expressed in the urinary sediment, we identified 39 miRNA targets that have significantly different expression between IgAN and CTL groups (14 up-regulated, and 25 down-regulated) (Table 3).

**Table 3:** Differential expression of urinary miRNA between IgA nephropathy and healthy control

up-regulated				down-regulated			
miRNA target	log of change	average expression	*P* value	miRNA target	fold of change	average expression	*P* value
hsa-miR-16^a^	58.234	7.250	0.0046	hsa-miR-204^a^	−8.523	4.019	0.0020
hsa-miR-26a^a^	53.378	7.183	0.0001	hsa-miR-431^a^	−6.362	4.015	0.0039
hsa-let-7 g	32.064	6.155	0.0000	hsa-miR-555^a^	−6.008	4.043	0.0039
hsa-miR-150^a^	30.220	5.854	0.0001	hsa-miR-137	−5.383	4.323	0.0037
hsa-miR-15a	17.288	6.650	0.0002	hsa-miR-34b	−4.597	5.016	0.0001
hsa-miR-423-3p	11.657	3.543	0.0002	hsa-miR-30c	−4.220	5.330	0.0020
hsa-miR-193b	11.311	4.411	0.0013	hsa-miR-542-3p	−4.062	5.733	0.0003
hsa-miR-155^a^	10.682	4.430	0.0028	hsa-miR-188-5p	−3.913	5.010	0.0034
hsa-miR-151-5p	10.277	3.361	0.0032	hsa-miR-615-5p	− 3.841	5.715	0.0003
hsa-miR-556-3p	8.282	3.050	0.0034	hsa-miR-518f	−3.789	5.428	0.0004
hsa-miR-361-5p	7.875	2.977	0.0047	hsa-miR-30a	−3.766	6.280	0.0004
hsa-miR-378	7.698	5.105	0.0018	hsa-miR-335	−3.744	5.000	0.0025
hsa-miR-221^a^	6.236	6.752	0.0014	hsa-miR-526a	−3.730	5.541	0.0004
hsa-miR-362-5p	6.223	4.511	0.0046	hsa-miR-300	−3.682	5.011	0.0031
				hsa-miR-142-5p	−3.611	5.271	0.0001
				hsa-miR-574-5p	−3.555	4.864	0.0003
				hsa-miR-651	−3.519	5.745	0.0005
				hsa-miR-206	−3.506	6.205	0.0009
				hsa-miR-1283	−3.506	6.864	0.0005
				hsa-miR-655	−3.435	5.181	0.0014
				hsa-miR-1206	−3.286	5.545	0.0007
				hsa-miR-708	−3.196	5.629	0.0035
				hsa-miR-10a	−3.058	6.646	0.0011
				hsa-miR-361-3p	−2.999	5.217	0.0005
				hsa-miR-548 g	−2.485	5.995	0.0041

^a^selected for second round validation

Of the 39 potential targets identified, 8 were chosen for further validation. Specifically, miR-16, miR-26a, and miR-150 were the three most up-regulated targets, miR-204, miR-431, and miR-555 were the three most down-regulated ones, miR-221 is the only target that was significantly up-regulated in the IgAN group as well as correlated with estimated GFR, and miR-155 has been reported by our previous study [22].

### Urinary miRNA level in the validation cohort

**Fig. 2:**
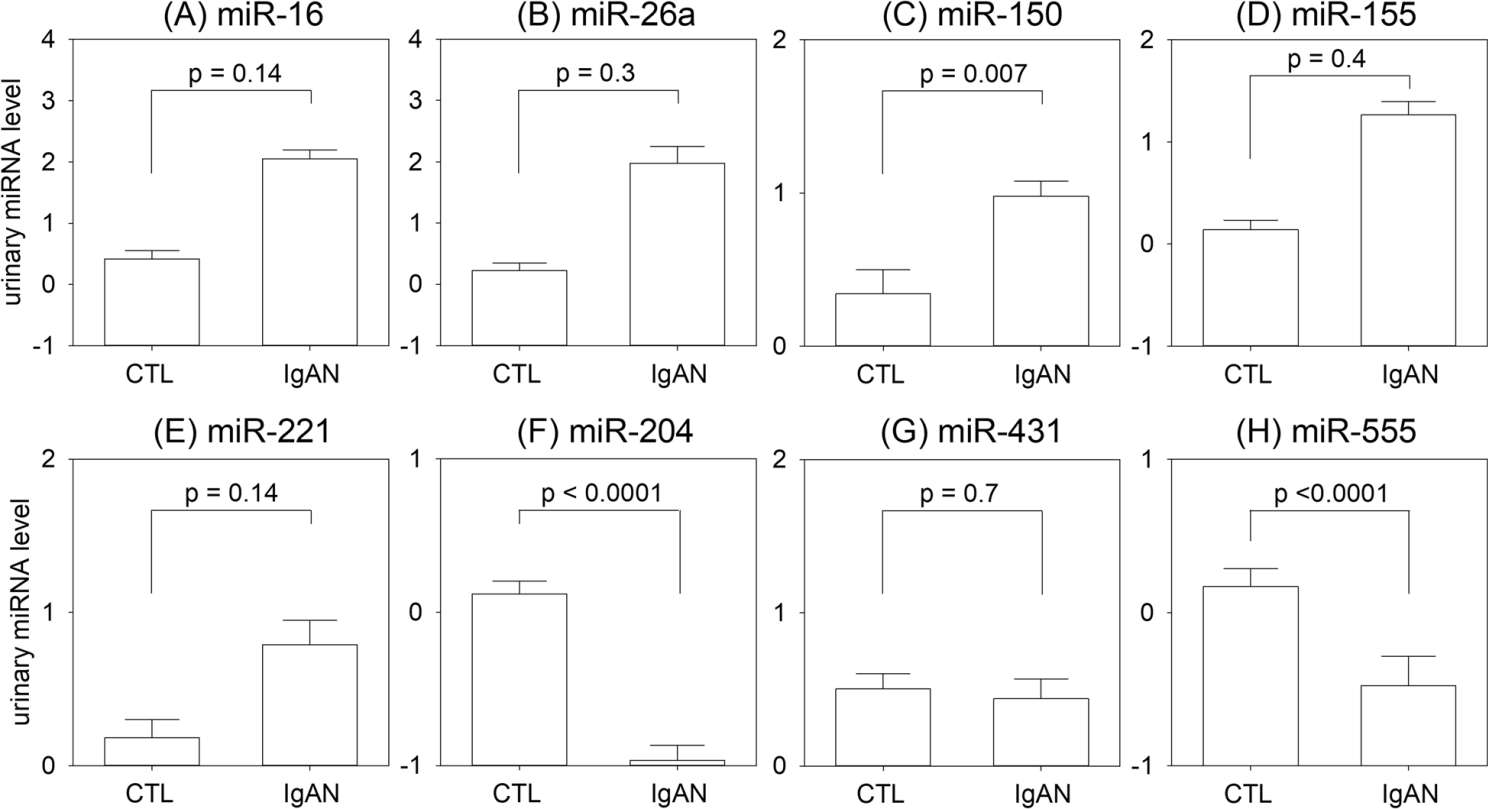
Comparison of urinary miRNA levels between IgA nephropathy (IgAN) and control (CTL) groups in the validation cohort: (**a**) miR-16; (**b**) miR-26a; (**c**) miR-150; (**d**) miR-155; (**e**) miR-221; (**f**) miR-204; (**g**) miR-431; and (**h**) miR-555. Whisker-box plot, with boxes indicate median, 25th and 75th percentiles, whiskers indicate 5th and 95th percentiles. Data are compared by Kruskal Wallis test

In the validation cohort, there were close internal correlation between urinary levels of miR-16, miR-26, miR-155 and miR-221 (Additional file 1: Table S1). The urinary miRNA levels of the IgAN and CTL groups in the validation cohort are summarized and compared in Fig. 2. In essence, urinary miR-204, miR-431 and miR-555 remained significantly reduced, but only urinary miR-150 level was significantly increased in the IgAN group as compared to the CTL group. In the validation cohort, the median increase in urinary miR-150 was 1.44 log (IQR − 0.21 to 2.12, *p* = 0.007); the median decrease in urinary miR-204, miR-431 and miR-555 were − 5.42 log (IQR − 3.25 to − 8.28, *p* < 0.0001), − 4.06 log (IQR − 0.34 to − 5.06, *p* = 0.023), and − 5.17 log (IQR − 2.40 to − 8.46, *p* < 0.0001), respectively.

### Accuracy of diagnosis

**Fig. 3:**
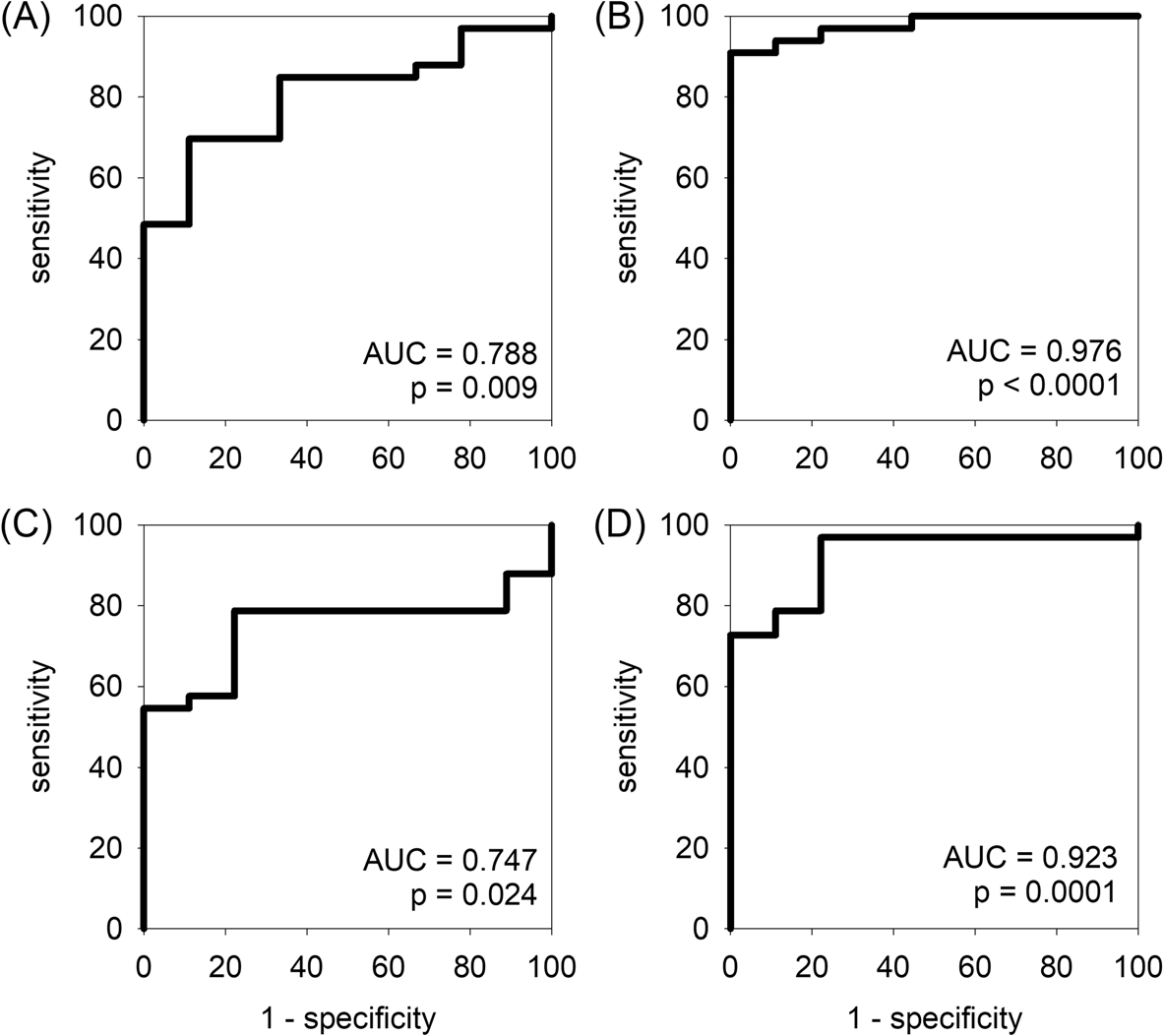
Receiver operating characteristic (ROC) curves indicating the performance of urinary levels of (**a**) miR-150; (**b**) miR-204; (**c**) miR-431; and (**d**) miR-555 for the diagnosis of IgA nephropathy. AUC, area under curve

The performance of urinary miRNA levels for the diagnosis of IgA nephropathy was explored by ROC curves (Fig. 3). In essence, the areas under curve (AUC) of the ROC curves of all 4 targets (miR-150, miR-204, miR-431, and miR-55) were statistically significant, with the AUC of miR-204 the highest (AUC = 0.976, *p* < 0.0001), and the diagnostic performance of combining additional miRNA targets was not further improved. For miR-204, an urinary level below 0.34 unit has a 100% specificity (and 90.9% sensitivity) in diagnosing IgA nephropathy, while the level above 1.70 unit has a 100% sensitivity (and 55.5% specificity) to exclude the diagnosis.

### Relation with clinical and pathological parameters

There were modest but statistically significant correlations between urinary miR-555 level and the duration of disease (Spearman’s r = − 0.590, *p* = 0.004) and baseline estimated GFR (r = 0.478, *p* = 0.024), but not with baseline proteinuria or any histological parameter. Urinary levels of miR-150, miR-204, miR-431, or other miRNA targets had no significant correlation with any baseline clinical or pathological parameter (details not shown).

The trend of renal function was observed for an average of 84.8 ± 30.2 months. None of the urinary miRNA targets correlated with the rate of estimated GFR decline or predicted the development of renal event (details not shown).

## Discussion

In the present study, we identified 4 miRNA targets whose urinary levels are significantly different between IgA nephropathy and healthy controls, and urinary miR-204 level alone has the best diagnostic accuracy.

Several previous studies explored the alterations in urinary miRNA levels in IgA nephropathy [12, 13, 22]. For example, Wang et al. [22] reported that intra-renal and urinary levels of miR-146a and miR-155 were significantly elevated in IgA nephropathy, and the degree of upregulation correlates with the severity of histological damage. Urinary miR-29b, miR-29c and miR-93 levels correlated with the clinical disease severity of IgA nephropathy and with the down-stream signaling of transforming growth factor-beta (TGF-β) pathway [13], indicating that they may play important roles in the pathogenesis of renal fibrosis. In another study, urinary miR-200a, miR-200b and miR-429 levels were reduced in IgA nephropathy, and the degree of reduction correlated with the rate of renal function deterioration [12]. Similarly, Min et al. [23] reported a significant difference in urinary exosomal miRNA profiles between patients with IgAN and healthy controls, and miR-29c, miR-146a and miR-205 may serve as novel non-invasive biomarkers for IgAN, while Liang et al. [24] found that urinary miR-21 and miR-205 levels are prognostic markers for evaluating the tubulointerstitial damage of IgAN. However, it is important to note that these studies used a candidate gene approach and lacked a second validation cohort. Our present study used a hypothesis-free approach that screened for all possible miRNAs in the urinary sediment, and the results are further validated by an additional independent cohort. It is interesting to note that in our development cohort, urinary miR-146a, miR-155, miR-29b, miR-29c and miR-93 levels were also significantly different between IgAN and CTL groups, but the difference did not reach the pre-defined criteria for the second stage validation. In contrast, urinary miR-200a, miR-200b and miR-429 levels were similar between IgAN and CTL groups in our development cohort.

Our result must be distinguished from the study of Serino et al. [6], which used high-throughput miRNA profiling and identified 37 miRNAs that were differentially expressed in the peripheral blood mononuclear cells (PBMC) of patients with IgA nephropathy as compared to healthy persons. In that study, PBMC miR-148b is upregulated in IgA nephropathy; miR-148b targets the enzyme core 1, β1,3-galactosyltransferase-1, modulates IgA1 O-glycosylation as well as the levels of secreted galactose-deficient IgA1 [6]. Our present study focused on the miRNA levels in urinary sediment rather than PBMC, and the alteration in miRNA levels is expected to be different. In our development cohort, urinary miR-148b level was marginally reduced in the IgAN group (0.84 log as compared to the CTL group, *p* = 0.034), but the difference did not reach the pre-defined criteria for the second stage validation.

Since the primary objective of our study is to identify an “miRNA signature” in urine for the diagnosis of IgA nephropathy, we did not explore – and there may not be any – biological role of the miRNAs that we found. As a result, we excluded patients with crescentic changes in the kidney biopsy and those with IgA vasculitis, so that a risk stratification based on urinary miRNA level is not possible. Nonetheless, previous studies showed that miR-204 takes part in the regulation of epithelial-mesenchymal transition by targeting SP1 in the tubular epithelial cells after ischemia-reperfusion injury [25], and in the control of local inflammation by regulating interleukin-6 (IL-6) receptor expression [26]. It remains to be determined whether these molecular pathways contribute to the pathogenesis of IgA nephropathy. Since the clinical spectrum of IgAN varies from minor urinary abnormalities to rapidly progressive renal failure, it may also be useful to clusterize patients with similar clinical phenotypes in further studies so as to identify miRNA for risk stratification.

There are a number of inadequacies in our study. First, the sample size is small and all of the patients came from a single center and were ethnically Chinese. It remains to be determined whether our result could be extrapolated to other patient population. This consideration is particularly important in the context of IgA nephropathy because its prevalence and clinical behavior are highly variable in different countries [27].

Second, healthy subjects served as control in the present study, but the healthy controls we recruited may not be well-matched with the patients (for example, in terms of age, renal function, or proteinuria), and, because of the difference in the number of subject, we could not perform a match-paired analysis for detailed comparison. More importantly, it remains uncertain whether the observed alterations in urinary miRNA levels represent disease-specific changes for IgA nephropathy or non-specific alterations secondary to renal scarring. It is absolutely important to conduct further studies that compare urinary miRNA level between IgAN and other glomerulonephritis in order to exclude the potential identification of damage-related miRNAs.

We did not determine the cellular origin of the miRNAs that we found in the urinary sediment. Previous studies suggested that both renal tubular cells and local inflammatory cells contribute to the total RNA in urine [10, 28]. Since the primary objective of our study is to identify urinary miRNA for diagnosis in routine clinical practice, we believe the cellular origin of miRNA may not be immediately relevant. Recent studies actually showed that conventional urinary sediment consists of cellular debris and microvesicles, and the miRNA level of these two components may be different [15, 29]. Further studies are needed to determine the miRNA alterations in various specific urinary components, which may further refine the accuracy of this novel non-invasive diagnostic tool.

## Conclusions

Urinary miR-150, miR-204, miR-431 and miR-555 levels are significantly different between IgAN and healthy controls; urinary miR-204 level alone has the best diagnostic accuracy. Our result suggests that urinary miRNA measurement could be developed as the diagnostic tool for specific glomerular diseases.

## Additional file

Additional file 1: Internal correlation of urinary miRNA levels in the validation set. (DOCX 16 kb)
