# Supplementary material for: Urinary miRNA profile for the diagnosis of IgA nephropathy
Source: BMC Nephrol. 2019 Mar 4;20:77. doi: 10.1186/s12882-019-1267-4 (PMC6399975; doi:10.1186/s12882-019-1267-4)
Supplement: Supplementary file 1 — Internal correlation of urinary miRNA levels in the validation set. (DOCX 16 kb) [file 12882_2019_1267_MOESM1_ESM.docx]

# Supplementary Table 1. Internal correlation of urinary miRNA levels in the validation set.

|  | miR-16 | miR-26a | miR-150 | miR-155 | miR-221 | miR-204 | miR-431 |
| --- | --- | --- | --- | --- | --- | --- | --- |
| miR-26a | r = 0.611,  p < 0.0001 |  |  |  |  |  |  |
| miR-150 | r = 0.630, p < 0.0001 | r = -0.134, p = 0.5 |  |  |  |  |  |
| miR-155 | r = 0.867, p < 0.0001 | r = 0.639,  p < 0.0001 | r = 0.423, p = 0.018 |  |  |  |  |
| miR-221 | r = 0.600,  p < 0.0001 | r = 0.557,  p < 0.0001 | r = 0.019, p = 0.9 | r = 0.767,  p < 0.0001 |  |  |  |
| miR-204 | r = -0.407, p = 0.01 | r = -0.537, p < 0.0001 | r = -0.133, p = 0.5 | r = -0.259, p = 0.12 | r = 0.047, p = 0.8 |  |  |
| miR-431 | r = -0.263, p = 0.3 | r = -0.615, p = 0.004 | r = -0.536, p = 0.04 | r = -0.403, p = 0.08 | r = 0.483, p = 0.03 | r = 0.268, p = 0.3 |  |
| miR-555 | r = -0.307, p = 0.1 | r = -0.709, p < 0.0001 | r = -0.041, p = 0.9 | r = -0.239, p = 0.2 | r = 0.049, p = 0.8 | r = 0.802, p < 0.0001 | r = 0.491, p = 0.053 |
